# Automated STI/HIV risk assessments: Testing an online clinical algorithm in Ottawa, Canada

**DOI:** 10.1177/09564624211031322

**Published:** 2021-09-10

**Authors:** Patrick O’Byrne, Alexandra Musten, Lauren Orser, Scott Buckingham

**Affiliations:** 1 School of Nursing, 6363 University of Ottawa, Ottawa, ON, Canada; 2 269770 Ontario HIV Treatment Network, Toronto, ON, Canada; 3 Machine, Ottawa, Canada

**Keywords:** Screening, HIV, sexually transmitted infections, self-testing, online testing

## Abstract

Despite the ongoing transmission of sexually transmitted infections (STIs) and HIV, many people became unable to access testing due to COVID-19. To address this, we created a mail-out HIV self-test kit, which could be delivered without restrictions in our region. The uptake and feedback from this project made us realize that comprehensive STI testing was being sought. To ensure testing occurred correctly—that is, it would be targeted at the persons most affected by STIs/HIV—we automated clinical decision-making. We built this model based on a 2-by-2 matrix that plots the risk of STI/HIV transmission and risk of STI/HIV exposure. The intercept of these two measures classifies a person as low, medium, or high risk. After automating this logic, 16 expert clinicians in STI/HIV care tested this system with over 400 test patient cases and refined the algorithm until it yielded the exact outcomes that these clinicians would offer patients based on guidelines. Findings of interest are that the scale of the *y*-axis is exponential, in that risk factors for exposure do not climb cumulatively but do so according to a quadratic equation. This helps ensure that testing services are targeted at those who are most inequitably burdened by these infections.

## Introduction

Across Canada and the United States, over the last 10 years, the rates of most bacterial sexually transmitted infections (STIs) have increased, while those for HIV have remained relatively stable.^1^ These infections, with little change, also continue to disproportionately affect the same, often minority, subgroups. In Ottawa, Canada, where this study is based, for HIV, the most affected populations include gay, bisexual, and other men who have sex with men (gbMSM); persons of African, Caribbean, or Black (ACB) ethnicities; persons who use drugs; trans persons; and members of Indigenous communities.^2^ For chlamydia, most infections are diagnosed in persons under 30 years of age, more often in females than males.^3^ gbMSM are also unequally burdened by gonorrhea,^4^ with research suggesting that up to 70% of such infections in gbMSM are in the oropharynx and rectum, not the genital tract.^5^ Syphilis, lastly, is mostly diagnosed among gbMSM,^6^ although recent increases among females^7^ suggest changes in epidemiology.

One prevention technique for STIs/HIV is testing, which identifies infections and connects people with care.^8–10^ For bacterial STIs, treatment eradicates infection, while for HIV, treatment can yield viral load suppression and a state of virtual non-infectivity.^11^ Following a status neutral approach,^12^ when testing yields negative results, other prevention techniques, such as counseling, pre-exposure prophylaxis (PrEP), and condoms, can mitigate risk.

Many people, however, do not access care for reasons related to distance, timing of services, wait times, cost, or fear of stigma (by clinicians and related to being seen seeking testing).^13–15^ Research also consistently identifies that one-third to one-half of gbMSM do not disclose their sexual orientation and practices to their primary care providers due to concerns about how practitioners might react.^16,17^ Another barrier is that clinicians may be unaware of how or when to offer testing, resulting in missed opportunities for earlier diagnoses or failed identification of extragenital infections.^18,19^ The outcome is that some patients either do not obtain testing or receive incomplete testing when they seek care—due to both personal apprehensions and health systems failures. As always, these barriers are more pronounced for minority groups.^20^ Unsurprisingly, the COVID-19 pandemic worsened access for STI/HIV care. In our jurisdiction, sexual health clinics closed walk-in services and screening for asymptomatic persons, which broadly resulted in an approximately 75% reduction in HIV testing.

To address this myriad of access issues, we developed GetaKit.ca,^21^ where persons can create an account, complete an STI self-assessment, and obtain STI/HIV screening based on reported practices. Our hope was that this system would promote STI/HIV testing both related to the COVID-19 pandemic and ongoing barriers to care, and would build on research that computer-assisted interviews yield more truthful answers regarding STI risk practices, compared to clinician-obtained histories.^22,23^ We also hoped that GetaKit would promote testing among visible and sexual minorities, although research^24–26^ suggests that online systems may be under-utilized by members of racialized communities. Nevertheless, we hoped that, even if a targeted outreach and a simple interface could not address this barrier, then GetaKit might at least streamline services for other groups, thus freeing up limited in-person clinician-time to provide services to minority groups. To implement GetaKit, we obtained funding from the Ontario HIV Treatment Network and research ethics approval from the University of Ottawa (H-02-20-5518). All participants who have used GetaKit have provided expressed consent for research and online services.

Unique to our project was that we created an algorithm which (1) stratifies participants based on reported risk practices and (2) recommends testing based on clinical guidelines.^27,28^ That is, we created an algorithm that automates STI/HIV clinical decision-making and which recommends specific tests to individual participants based on their reported information. While other online risk calculators exist, these often only stratify persons’ level of risk, whereas our system recommends and provides direct access to relevant STI/HIV testing.^29,30^ In this article, we report on our algorithm and demonstrate its functionality using five archetypal patients which show the algorithm’s responsiveness to varying risk profiles. These cases also highlight how our algorithm could help ensure that members of the groups most affected by STIs/HIV receive comprehensive testing.

## Understanding STI/HIV risk assessments

The task of STI/HIV clinicians is to determine which parts of patients’ anatomy were involved in sexual contact, when contacts occurred, and what were the characteristics of partners.^8^ This includes inquiries about oral, vaginal, and anal sex; about prevention strategies (e.g., condoms and PrEP); about the sex/gender and ethnicity of partners; and other risk practices (e.g., injection drug use and sex work). (Table 1.) Then, clinicians analyze collected data to determine risk.

**Table 1.** STI/HIV risk assessment questions.

Question category	Sub-questions
Demographics	Age
	Sex
	Sexual orientation
	Gender
	Ethnicity
	Country of birth
Sex practices	Oral, vaginal, anal, sex toys
Sex partner characteristics	Sex and gender
	ACB
	Born in countries where HIV is endemic
	Bisexual
	Injection drug use
	Sex work
Other risk practices	Personal injection drug use
	Sex work
STI/HIV history	When last tested
	New partners since last tested
	Prior diagnoses

STI: sexually transmitted infection; ACB: African, Caribbean, and Black.

**Figure 1.**
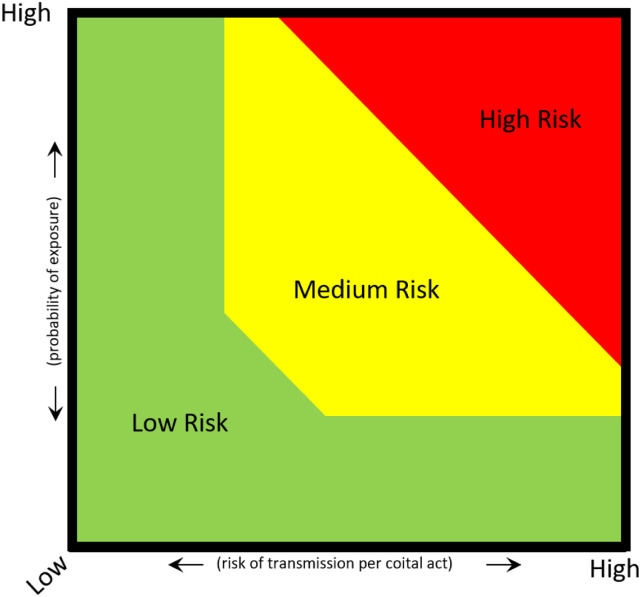
STI/HIV risk matrix. STI: sexually transmitted infection.

Figure 1 illustrates this clinical risk assessment process with a two-by-two matrix, which has a person’s reported risk practices and associated risk of transmission on the *x*-axis and their probability of exposure^1^ to a given STI on the *y*-axis. As one moves along the *x*- and *y*-axes, risk varies. To determine risk, clinicians must obtain information about each axis and plot the intercept.

**Figure 2.**
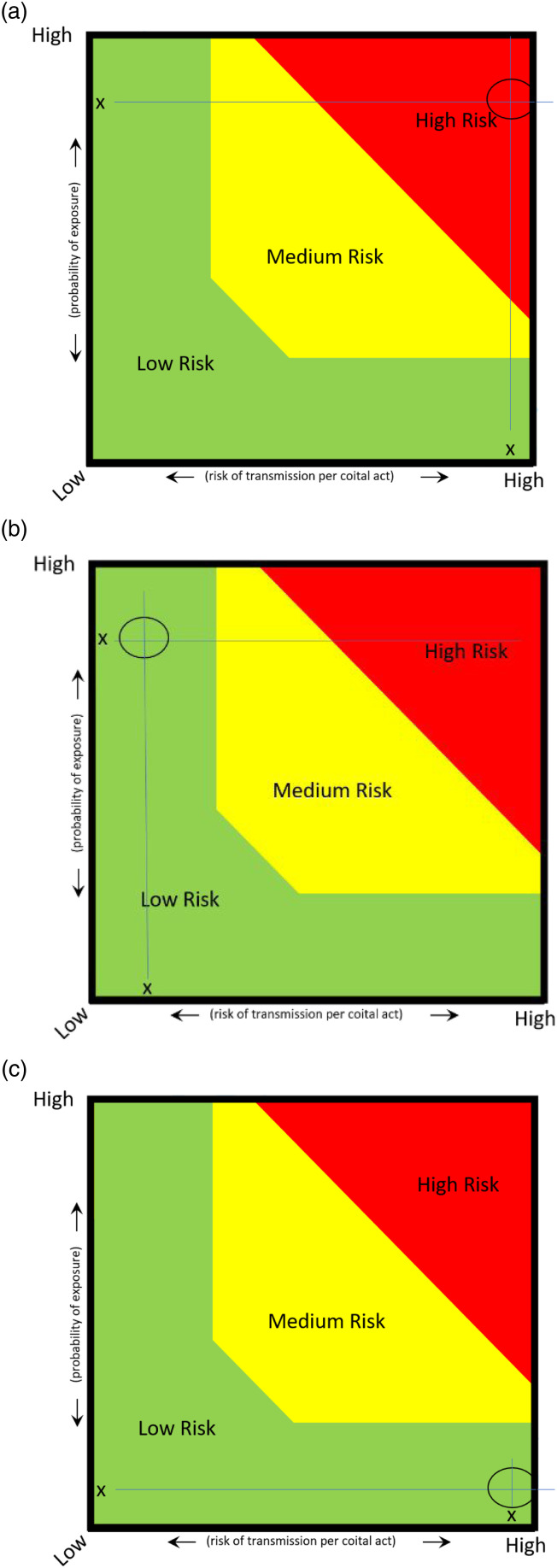
(a) Examples of STI/HIV risk assessments.

To explain further, we can use the case of a gay man who neither uses condoms nor PrEP and engages in receptive anal sex. For HIV, these practices put him at the far right of the *x*-axis and his same-sex partners put him at the top of *y*-axis. Plotting this makes him “high risk.” (Figure 2(a).)

If, however, this same person only engages in oral sex with male partners, the risk of HIV transmission is low, even if the probability of having an HIV-positive partner is high. (Figure 2(b).)

Similarly, a white Canadian-born 20-year-old female who engages in condomless vaginal sex with white male partners is low risk for HIV (because while the risk of transmission is high, the probability of exposure is low—Figure 2(c)); she would, however, be high risk for chlamydia (as the prevalence is high among this demographic—Figure 2(a)).

This matrix can, therefore, determine overall risk by plotting exposure probability and risk of transmission. (For more information on this matrix, please see O’Byrne et al.^38^). The limitation though is that clinicians must know the risk of transmission for all sexual practices and the prevalence of STIs/HIV in many populations. While this may be possible in STI clinics, it is less possible in primary care. Other barriers to testing (as noted above) further undermine access to testing and limit the effectiveness of testing for STI/HIV prevention initiatives.

## The algorithm

### Building the algorithm

Due to higher-than-expected uptake^21^ ^2^ for our HIV self-testing project and profound restrictions on access to STI/HIV testing due to the COVID-19 pandemic, we worked to offer full STI testing via our online platform. This involved three steps. First, we engaged in community consultations with local gbMSM, ACB, Indigenous, and trans organizations to create culturally sensitive and trauma-informed questions that would be non-stigmatizing for participants. As part of this, we built a 20-question STI self-assessment that participants could complete via GetaKit.ca. These questions inquired about all items in Table 1. As part of this self-assessment, participants were encouraged to seek in-person care if they reported symptoms, were a contact of an STI or HIV, or required post-exposure prophylaxis (PEP) or emergency contraception. We also reviewed STI/HIV testing window periods and encouraged retesting based on reported timelines. The logic to our model regarding window periods was to “test and retest” to identify infections that pre-existed the last reported sexual contact. We identified the utility of this approach in our PEP study,^31^ where some participants who presented for PEP had undiagnosed HIV infections.

Second, we reviewed the GetaKit self-assessment with stakeholders who worked in the field of STI/HIV testing and prevention and modified the language and questions accordingly. This phase did not involve research participants, but peers who worked for partner agencies. Our goal was to have knowledgeable peers in community organizations refine our self-assessment.

Third, we converted Figure 1 into an algorithm that a computer could use to impute a risk score based on the reported data from Table 1 for the following infections: HIV, syphilis, hepatitis C, and gonorrhea and chlamydia for all anatomical sites where one could acquire these infections (oropharynx, rectum, vagina, and urethra). This involved creating scores for both the *x*- and *y*-axes and numerical thresholds for tests to be recommended. Our calculation was simple: sum the risk score, sum the population score, multiply these scores, and determine if these outcome values breached the test threshold. We created this algorithm in Google Sheets, which was sufficiently robust for our needs. Indeed, because our model stratifies participants based on their risk profiles and risk practices, we were not predicting and did not need more advanced software.

**Figure 3.**
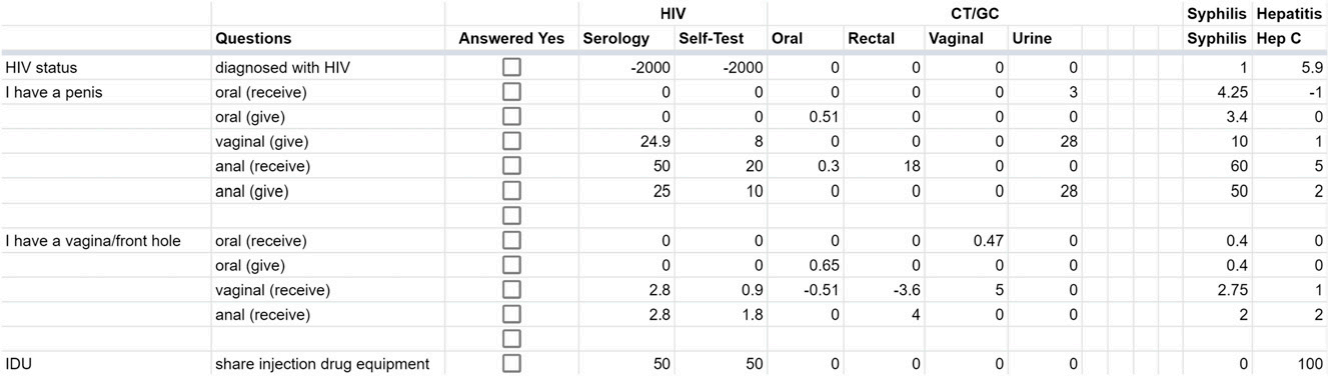
STI/HIV risk scores.

For the *x*-axis, we used established risk levels from the research for the probability of STI/HIV transmission for different practices to stratify practices as low, medium, or high risk.^32–34^ We determined that the scores for a low-risk practice were <1, for medium-risk practice ranged from 1 to 9, and for a high-risk practice were ≥10. Items such as HIV-status, PrEP, and condom use further adjusted the risk scores. (Figure 3.) The variation in the assigned scores allowed our algorithm to trigger specific testing for certain practices in isolation (e.g., injection drug use and hepatitis C testing) or only when a set of risk practices were reported in combination (e.g., receptive anal sex and male gender for rectal gonorrhea/chlamydia testing).

**Figure 4.**
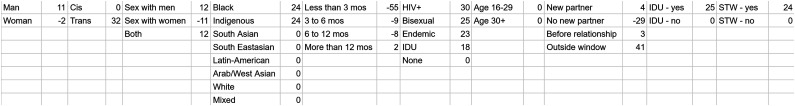
Population scores.

For the *y*-axis, we weighted populations based on local prevalence, with low prevalence groups having a score ≤0, medium prevalence groups having a score ranging between 1 and 11, and high prevalence groups having a score ≥12. For population variables, we included age, sex, gender, ethnicity, sex of partners, characteristics of sex partners (ACB, HIV-positive, IDU), personal use of illicit drugs, engagement in sex work, last time tested, and if the participant reported new sexual partners since their last STI/HIV testing. For each infection, the scores attached to each of the foregoing items were combined to generate a final population score. Figure 4 shows an example of this weighting for HIV.

### Testing the algorithm

To operationalize the algorithm, we had the computer sum the risk scores for practices (*x*-axis) and exposure (*y*-axis) and multiply these to determine the final score. This means that, were a person’s reported practices to include condomless receptive and penetrative anal sex, the formula dictated that the computer would add the score for engaging in receptive anal sex (a high-risk score for HIV) with the score for penetrative anal sex (a high-risk score for HIV) to yield the final score for HIV testing. As can be seen from Figure 3, the cumulative risk score for HIV serology for the foregoing practices in someone with a penis would be 75 points. The same process occurred for all reported practices for each infection. The output of this calculation was then multiplied by the total population score and compared to the appropriate test threshold. The algorithm then recommended tests when the test threshold was exceeded. While the risk matrix classified risk as low, medium, or high, the testing threshold was set at a medium risk.

At first, this process did not yield results that corresponded with recommended STI/HIV testing. We attempted to correct this by varying the test thresholds, but this did not yield the desired outcomes. Through further development, we realized that increasing population risk scores exponentially rectified this situation. That is, we determined that two population risk factors did not sum as 1 + 1 = 2, but rather, increased in larger steps based on ranges. A population score of ≤2 was multiplied by 0 and a score of ≥3 was transformed using the following equation:

$$y=0.0001x^{3}+0.356x^{2}+0.0978x-0.3615$$

Transformation of the population scores according to this formula corresponded with testing recommendations that more closely aligned with guidelines. To further refine this, we adjusted the weights assigned to each sexual practice until appropriate testing was recommended in all fictitious evaluation cases that were input by our evaluation team of specialized healthcare professionals who worked in our local STI clinic. Indeed, we refined the algorithm by having a team of three physicians, three nurse practitioners, and ten registered nurses input over 400 fictitious test cases to ensure results corresponded with clinical guidance documents for STI/HIV testing. After including the exponential multiplication of population risk scores before multiplying the test score by the population score and slight adjustments in the assigned risk score for varying sexual practices, the outcome was perfect alignment between the algorithm’s recommendations and Public Health Agency of Canada and Public Health Ontario clinical practice guidelines.^27,28^

## Clinical examples

To demonstrate the STI/HIV algorithm, we will show the scoring process for five exemplar cases. These encompass a range of screening situations and highlight the responsiveness of the algorithm to varying practices and participant characteristics.

### Case 1

The first case is an HIV-negative, white, cis-gendered, 40-year-old male who engages in oral sex (received/performed) and anal sex (received/performed) with male partners. He does not use condoms or PrEP. He was last tested for STIs/HIV about 6 months ago and had a new sexual partner since. He is unsure if any of his sexual partners engage in injection drug use and does not report doing so himself, nor does he report sex work. Based on these practices, clinical guidelines would indicate that this person should receive HIV testing (serology and/or a point-of-care test), syphilis serology, and gonorrhea and *chlamydia* testing by urine and by oral and rectal swabs. The algorithm correctly identified these recommended tests. See Box 1.

**Box 1.** 

Testv	Test required	Final score	Pop. multiplier	Pop. score	Question score
HIV serology	Yes	1125	15	19	75
HIV self-test	Yes	450	15	19	30
Oral	Yes	85.455	105.5	53	0.81
Rectal	Yes	1899	105.5	53	18
Vaginal	No	0	105.5	53	0
Urine	Yes	3270.5	105.5	53	31
Syphilis	Yes	1411.8	12	17	117.65
Hep C	No	0	0	−9	6

Of note, if this person were to report being HIV-positive, then HIV testing is removed. See Box 2.

**Box 2.** 

Test	Test required	Final score	Pop. multiplier	Pop. score	Question score
HIV serology	No	−28875	15	19	−1925
HIV self-test	No	−29550	15	19	−1970
Oral	Yes	85.455	105.5	53	0.81
Rectal	Yes	1899	105.5	53	18
Vaginal	No	0	105.5	53	0
Urine	Yes	32705	105.5	53	31
Syphilis	Yes	1423.8	12	17	118.65
Hep C	No	0	0	−9	11.9

### Case 2

The second case involves an HIV-negative, white, cis-gendered, 20-year-old female with male partners. She reports condomless vaginal sex and oral sex (receive/perform). She does not report sex work or injection drug use. She reports that her partners are white and do not use injection drugs either. She was last tested 6 months ago for STIs/HIV and has had new sexual partners since. Based on the guidelines, this female should have a vaginal gonorrhea/*chlamydia* test only. While oral testing could be indicated, local guidelines do not recommend this in the absence of a “clinical indication.” HIV and syphilis testing are also only recommended annually for this person. The algorithm corresponded exactly with such recommendations. See Box 3.

**Box 3.** 

Test	Test required	Final score	Pop. multiplier	Pop. score	Question score
HIV serology	No	4.2	1.5	6	2.8
HIV self-test	No	1.35	1.5	6	0.9
Oral	No	8.925	63.75	39	0.14
Rectal	No	−229.5	63.75	39	−3.6
Vaginal	Yes	348.7125	63.75	39	5.48
Urine	No	0	63.75	39	0
Syphilis	No	12.425	3.5	9	3.55
Hep C	No	0	0	−11	1

### Case 3

The third case is an HIV-negative, Black, cis-gendered, 26-year-old heterosexual female with male partners who are also Black. She reports condomless vaginal and anal sex and performing oral sex. She was last tested 6 months ago with new partners since. She does not report injection drug use for herself or her partners. Per local guidelines, this female should receive a vaginal gonorrhea/*chlamydia* test. Due to elevated HIV incidence among Black women, serology for HIV and a rapid HIV test should also be offered. No increased screening is warranted for syphilis. The algorithm yielded these exact recommendations for this test case. See Box 4.

**Box 4.** 

Test	Test required	Final score	Pop. multiplier	Pop. score	Question score
HIV serology	Yes	210	37.5	30	5.6
HIV self-test	Yes	101.25	37.5	30	2.7
Oral	No	8.925	63.75	39	0.14
Rectal	No	25.5	63.75	39	0.4
Vaginal	Yes	348.7125	63.75	39	5.47
Urine	No	0	63.75	39	0
Syphilis	No	19.425	3.5	9	5.55
Hep C	No	0	0	−11	3

Notably, if this same person reports that she was last tested less than 3 months ago, but that she is now outside the testing window for HIV, the algorithm removes the gonorrhea and *chlamydia* testing while retaining the HIV testing to rule out infection at the appropriate time. See Box 5.

**Box 5.** 

Test	Test required	Final score	Pop. multiplier	Pop. score	Question score
HIV serology	Yes	92.4	16.5	20	5.6
HIV self-test	Yes	44.55	16.5	20	2.7
Oral	No	0		−166	0.14
Rectal	No	0		−166	0.4
Vaginal	No	0		−166	5
Urine	No	0		−166	0
Syphilis	No	0	0	−61	5.15
Hep C	No	0	0	−41	3

### Case 4

The fourth case is an HIV-negative, white, 32-year-old trans-male who has internal genitals. They engage in oral sex (performs only) and anal sex (receptive only) with partners who have external genitals. They report injection drug use and no sex work. They were last tested between 3 and 6 months ago and have had new partners since. According to current guidelines,^25,26^ trans-males are disproportionately affected by STIs/HIV and warrant comprehensive testing. Based on the identified risk practices, this person should receive gonorrhea and *chlamydia* testing (oral and rectal), syphilis, HIV, and hepatitis C serology, and an HIV rapid test. The algorithm identified such testing appropriately. See Box 6.

**Box 6.** 

Test	Test required	Final score	Pop. multiplier	Pop. score	Question score
HIV serology	Yes	295.4	105.5	75	2.8
HIV self-test	Yes	189.9	105.5	75	1.8
Oral	Yes	68.575	105.5	142	0.65
Rectal	Yes	422	105.5	142	4
Vaginal	No	0	105.5	142	0
Urine	No	0	105.5	142	0
Syphilis	Yes	169.2	70.5	41	2.4
Hep C	Yes	80	40	31	2

### Case 5

The last case involves an HIV-negative, Indigenous, cis-gendered 35-year-old female with male partners. She engages in vaginal and oral sex (receives/performs). She was last tested over 12 months ago and had a new partner 6 months ago. She does not report injection drug use for herself or her partners, nor does she report sex work. Per local guidelines,^27,28^ this person should receive gonorrhea/*chlamydia* testing (vaginal only), plus serology for HIV and syphilis. Due to elevated HIV and hepatitis C prevalence among members of Indigenous populations in Canada, this person should also be offered a rapid HIV test and hepatitis serology. The algorithm yields these outcomes. See Box 7.

**Box 7.** 

Test	Test required	Final score	Pop. multiplier	Pop. score	Question score
HIV serology	Yes	295.4	105.5	53	2.8
HIV self-test	Yes	94.95	105.5	53	0.9
Oral	No	14.77	105.5	59	0.14
Rectal	No	−379.8	105.5	59	−3.6
Vaginal	Yes	577.085	105.5	59	5.47
Urine	No	0	105.5	59	0
Syphilis	Yes	84.3125	23.75	24	3.55
Hep C	Yes	23.75	23.75	24	1

## Discussion

In response to a higher-than-expected uptake of our HIV self-test study^22^ plus profound reductions in access for STI/HIV testing due to COVID-19, we expanded Getakit.ca to include automated clinical decision-making for STI/HIV risk assessments. We believe this is the first of such algorithms that operates through a website and consistently recommends testing with a high degree of sophistication according to local guidelines^27,28^ based on participants’ reported risk practices. This algorithm raises a few noteworthy points for discussion.

First, the accuracy our algorithm is notable. Sixteen healthcare professionals from nursing and medicine who work in our STI clinic tested the system by inputting over 400 fictitious test cases with different risk practices and profiles and found that the algorithm output matched local clinical guidelines^27,28^ perfectly. The importance of this finding cannot be overstated. While accuracy ensures that people who seek testing can obtain such services, both generally and during pandemics, accuracy of testing distribution also ensures equitable allocation of finite resources to those who are most affected by STIs/HIV. This accuracy also ensures that persons are offered testing based on provincial and federal health guidelines,^27,28^ which has been a barrier to implementation of previous online testing programs in our jurisdiction. While access to testing is important, indiscriminate delivery can also produce unwanted outcomes. For example, testing without appropriate assessment can exacerbate inequitable healthcare delivery access. In the context of ongoing testing supply shortages, providing these resources to members of groups not burdened by STIs/HIV means that the members of the groups with the greatest burden might not have access to testing. Another issue is that all testing has performance limitations, which are magnified by prevalence. As the positive predictive value of a test decreases in tandem with prevalence, test accuracy decreases when persons who are unlikely to have infections are tested. In addition to issues regarding resources, such inappropriate testing can also generate ethical issues related to the consequences of false positive results, such as requiring treatment or partner follow-up, and any distress associated with diagnosis. Our automated STI/HIV risk assessment and screening algorithm helps rectify this situation by appropriately targeting testing.

A second noteworthy point about our algorithm is its malleability. Figures 3 and 4 show that each test and each population is weighted individually, allowing for the algorithm to be tailored based on changing epidemiology and research. If evidence emerges showing differing estimates of STI/HIV transmission risk or about new prevention technologies, the algorithm can be easily updated by re-allocating risk scores; the logic and calculations within the algorithm, however, remain unchanged. The layout we developed for the algorithm similarly allows it to function in diverse geographic settings, provided that the population scores are updated to reflect local epidemiology. The cumulative calculations for population scores also allow new risk groups or new risk factors to be added to score calculations, again without requiring major modifications to the automated process. To the best of our knowledge, no similar automated algorithms exist.

Third, the malleability and accuracy of our algorithm means that it could be used to increase STI/HIV testing uptake among those who most commonly avoid testing. While we would always consider in-person healthcare to be optimal, we know such interactions are problematic for many due to geography, wait times, and concerns about being seen.^13–16^ We also know that many persons avoid STI/HIV testing due to concerns about stigmatization, and that such concerns increase among persons who are trans, ACB, Indigenous, gbMSM, etc.^17,35–37^ In other words, accessing STI/HIV testing is often most difficult for the persons who are most affected by these infections. As such, a possible utility of our algorithm is that it can provide care in non-stigmatizing ways using culturally sensitive, non-judgmental language for persons who historically have had negative experiences with the healthcare system regarding their ethnicities, skin color, sexual orientation, or gender identities. This is a major strength to this algorithm.

Another strength of our algorithm is that it ensures that the members of the groups that are most affected by STIs and HIV can obtain full services. While research^24–26^ has shown that some algorithms formalize ethnic biases and consequently impede access to care for racialized communities, we designed ours so that the thresholds to qualify for care were more easily surpassed by members of these groups. In opposition to what has been found in some previous healthcare algorithms, therefore, we ensured easier access to care for minority and racialized groups; notably, this approach was supported by our community consultations.

### Limitations

The development and utility of our STI/HIV screening algorithm is not without limitations. Our work was based on Canadian guidelines only, with a specific focus on STI/HIV epidemiology in Ottawa. Its applicability more broadly has not been tested, although the algorithm is sufficiently nimble to allow for adjustments based on local prevalence data. Another limitation is that the algorithm requires field testing. Trained clinicians with in-depth understanding of the subject material completed the validation using fictitious patient scenarios. If such a high degree of alignment with guidelines will occur when the system is used by the lay public is yet to be determined. A reassuring finding though is that the clinicians who tested the system were not trained in how to register, navigate, or complete the self-assessment and test ordering, and all were able to complete the process.

## Conclusion

In this article, we presented an STI/HIV risk assessment algorithm that we developed in Ottawa, Canada and showed the logic we automated to stratify participants’ STI/HIV risk to ensure they were offered appropriate testing, in accordance with local guidelines. Our robust pilot testing with over 400 test patient cases showed the accuracy of our algorithm to recommend the same testing that our 16 expert STI/HIV clinicians would have offered. We believe this is the first of such algorithms to exist and posit that a major strength of our system is its ease of modification based on changing epidemiology and scientific evidence about STI/HIV transmission and prevention. While we believe that in-person testing is ideal, we think this automated online system might overcome some barriers to STI/HIV testing, especially for minority and marginalized persons who are both most burdened by STIs/HIV and often most victimized by the healthcare system. Mass roll-out and uptake evaluation will determine if this assertion holds true.

## Notes

1 The logic for the *y*-axis is that, as STI/HIV prevalence increases within a person’s sexual network, so does the risk of exposure (i.e., of having a sexual partner with an STI or HIV).

2 For our initial HIV self-test study, we can intended to test a minimum of 150 participants in the first 6 months of implementation. By 7 months, we had distributed 444 test kits.
